# Clinical efficacy of laparoscopic cholecystectomy combined with percutaneous transhepatic gallbladder drainage in severe acute cholecystitis: an analysis of prognostic risk factors

**DOI:** 10.3389/fsurg.2025.1609327

**Published:** 2025-06-26

**Authors:** Lexiang Chen, Mingfu Hu, Shanhu Huang, Yi Sun

**Affiliations:** Department of General Surgery, Yongjia County Traditional Chinese Medicine Hospital, Wenzhou, Zhejiang, China

**Keywords:** severe acute cholecystitis, laparoscopic cholecystectomy, percutaneous transhepatic gallbladder drainage, clinical efficacy, prognosis

## Abstract

**Objective:**

To analyze the clinical efficacy of laparoscopic cholecystectomy (LC) combined with percutaneous transhepatic gallbladder drainage (PTGBD) in patients with acute critical cholecystitis.

**Methods:**

One hundred patients diagnosed with severe acute cholecystitis were retrospectively selected and categorized into two groups based on the surgical approach: the joint group (*n* = 49, underwent LC combined with PTGBD) and the LC group (*n* = 51, underwent LC alone).

**Results:**

The joint group demonstrated a significantly shorter surgery duration and lower intraoperative blood loss compared to the LC group (*P* < 0.05). On the third postoperative day, patients in the joint group exhibited lower levels of WBC and CRP than those in the LC group (*P* < 0.05). The joint group showed faster recovery of bowel function, earlier ambulation, and shorter time to resume oral intake compared to the LC group (*P* < 0.05). Additionally, the joint group reported higher satisfaction than the LC group (*P* < 0.05). However, the joint group incurred higher surgical costs, total hospitalization costs, and medication costs than the LC group (*P* < 0.05). The independent risk factors for postoperative complications in patients with severe acute cholecystitis included a disease onset longer than 72 h, a surgical approach of LC alone, surgery duration longer than 2 h, intraoperative blood loss >100 ml, and age ≥65 years (*P* < 0.05).

**Conclusion:**

Compared with LC alone, LC combined with PTGBD is more effective in reducing surgical trauma in patients with severe acute cholecystitis, improving postoperative inflammatory markers, and accelerating recovery. However, this combined approach is associated with significantly higher direct medical costs during hospitalization.

## Introduction

Acute cholecystitis is a common acute abdominal condition in general surgery, with its incidence steadily rising. When complications such as gangrene or perforation occur, the condition may progress to severe acute cholecystitis, which can be life-threatening without timely intervention (1, 2). Laparoscopic cholecystectomy (LC) is the gold standard for treating gallbladder diseases due to its minimally invasive nature and rapid recovery (3). However, in case of severe acute cholecystitis, LC poses challenges including increased surgical difficulty, higher complication rates, and a greater likelihood of conversion to open surgery. Moreover, elderly patients or those with comorbidities may not tolerate LC, complicating clinical management (4).

Percutaneous transhepatic gallbladder drainage (PTGBD) offers a minimally invasive means to rapidly alleviate symptoms and reduce gallbladder pressure, thereby facilitating subsequent surgery (5). Recent studies (6, 7) suggest that combining PTGBD with LC can improve outcomes in severe cases by initially controlling inflammation and lowering surgical risk. This approach may also expand treatment options for high-risk patients.

Despite its promise, few studies have systematically analyzed the clinical efficacy of combining LC and PTGBD, particularly in relation to perioperative complications, recovery metrics, patient satisfaction, and cost-effectiveness. Addressing this gap, the present study evaluated key clinical outcomes, such as surgical indicators, inflammatory and liver function markers, complications, and satisfaction, between LC alone and LC combined with PTGBD. Additionally, logistic regression analysis was used to identify independent risk factors for postoperative complications, aiming to inform surgical decision-making and optimize perioperative management.

## Materials and methods

### Study design and patient selection

This retrospective study was approved by the ethics committee of Yongjia County Traditional Chinese Medicine Hospital. All patients and their families signed the informed consent form prior to the beginning of the treatment. Patient selection was conducted using the hospital's electronic medical record, based on the following inclusion criteria:

Inclusion criteria: (1) Age between 30 and 70 years; (2) Onset of symptoms <2 weeks; (3) Diagnosis of severe acute cholecystitis according to Tokyo Guidelines (8).

Initially, 153 patients met these criteria. Subsequently, a second screening based on the exclusion criteria was performed:

Exclusion criteria: (1) Severe dysfunction of liver, kidney, or other organs; (2) Concurrent hemorrhagic disorders or severe coagulation dysfunction; (3) ASA classification of III or higher; (4) Concurrent biliary malignancies; (5) Presence of gallbladder polyps; (6) Non-calculous cholecystitis with duodenal space-occupying mass or other complications; (7) Incomplete data.

**Figure 1:**
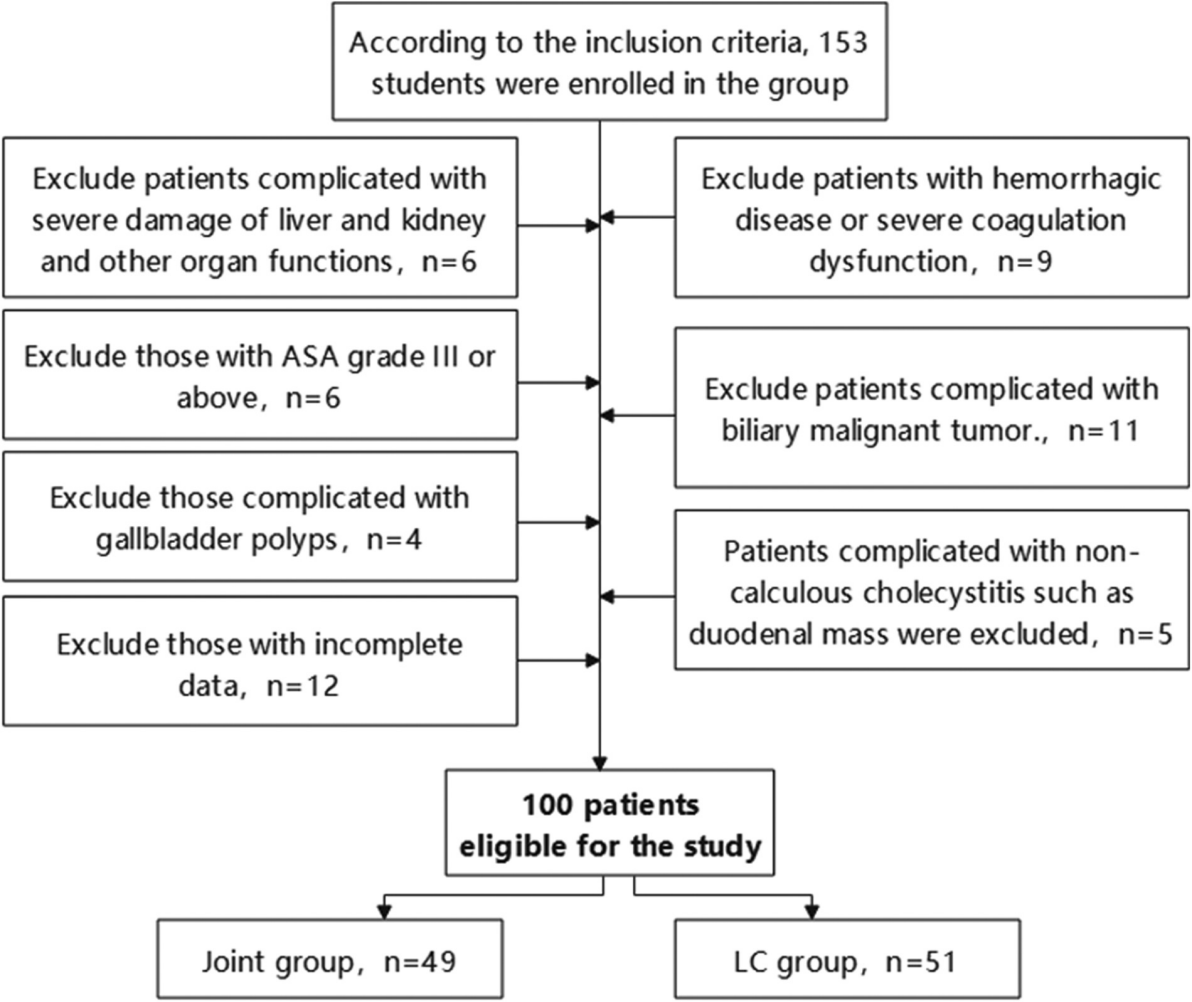
Flow diagram of patient inclusion and exclusion.

After screening, 100 patients were included in the study (Figure 1). Based on surgical approach, they were categorized into two groups: the joint group (*n* = 49, LC combined with PTGBD) and the LC group (*n* = 51, LC alone).

### Scheduling of combined procedures

Patients in the joint group (*n* = 49) first underwent PTGBD to control acute inflammatory response. Following PTGBD, patients entered an observation phase. Once clinical symptoms improved, inflammatory markers such as WBC and CRP declined, and ultrasound confirmed resolution of gallbladder wall edema, LC was performed. The average interval between PTGBD and LC was 7–14 days, determined based on the degree of inflammation resolution and results of preoperative evaluation. The primary criteria for proceeding with LC included: (1) Normalization of body temperature and either complete resolution or significant relief of abdominal pain; (2) Normalization or near-normalization of WBC counts; (3) A marked decrease in CRP levels compared with pre-PTGBD values; (4) Ultrasonographic evidence indicating substantial resolution of gallbladder wall edema. All LC procedures were performed by the same surgical team using standardized surgical techniques and protocols. Intraoperatively, the drain was removed concurrently with cholecystectomy in all patients. Adhesions between the gallbladder and the drain were carefully dissected first. The drain was then clamped, and the gallbladder was completely resected. No additional postoperative drain removal was required.

### Data collection

Data were collected from the hospital information system and included: (1) Baseline characteristics: sex, age, BMI, LYM, RBC, PLT, blood pressure, surgical history, alcohol consumption, hypertension, diabetes, etc.; (2) Surgical parameters: surgery duration, intraoperative blood loss, total hospital stay and postoperative stay; (3) Laboratory indicators: preoperative and 3-day postoperative inflammatory markers (WBC count and CRP levels) and liver function indicators (AST and ALT levels); (4) Perioperative complications: bile leaks, incision infections, etc.; (5) Postoperative recovery indicators: bowel function recovery, time to ambulation, and time to resume oral intake; (6) Patient satisfaction: assessed using a self-developed scale with a maximum score of 100, with higher scores indicating greater satisfaction; (7) Economic indicators: surgical costs, total hospitalization expenses, and medication costs.

### Outcome measures

#### Main outcome measures

The preoperative and postoperative inflammatory markers, liver function indicators, postoperative recovery, and perioperative complications were compared between the two patient groups. Logistic regression analysis was employed to identify independent risk factors influencing the occurrence of perioperative complications in patients with severe acute cholecystitis.

#### Secondary outcome measures

The basic surgical parameters, patient satisfaction, and economic indicators were compared between the two groups.

#### Application of results

In this study, a data separation approach was employed to minimize analytical bias. Data collection and data analysis were assigned to different personnel. Specifically, patient information was extracted from the electronic medical record system by designated data collectors and then anonymized using coded labels (e.g., Patient 1, Patient 2). The dataset was then reviewed by the project leader to ensure completeness before being transferred to independent data analysts for statistical analysis. Strict confidentiality measures were implemented throughout the process. All research team members received prior training on the appropriate use of clinical data to ensure compliance with relevant data protection and ethical standards.

#### Statistical methods

The data were processed with SPSS 21.0. The normality of continuous variables was assessed using the Shapiro–Wilk test. The results indicated that all continuous variables were normally distributed (*P* > 0.05); therefore, data were presented as mean ± standard deviation, and comparisons between groups were conducted using the independent samples *t*-test. In cases where data did not meet the assumption of normality, results were planned to be expressed as median (interquartile range, IQR), and comparisons between groups would be performed using the non-parametric Mann–Whitney *U* test. Categorical variables were expressed as percentages (%), and comparisons between groups were conducted using the chi-square (*χ*^2^) test or Fisher's exact test, as appropriate. Risk factor analysis was performed using logistic regression. To minimize the exclusion of potential risk factors, variables with *P* < 0.1 in the univariate analysis were included in the multivariable analysis. All graphs in this study were generated using GraphPad Prism 8.0. *P* < 0.05 was considered statistically significant.

## Results

### Comparison of baseline clinical data

Baseline characteristics, including sex, age, BMI, and medical history, showed no statistically significant differences between the two groups (*P* > 0.05). However, the proportion of patients in the joint group with a disease onset longer than 72 h was significantly higher than that in the LC group (*P* < 0.05, Table 1).

**Table 1:** Comparison of baseline clinical data between the two groups $\left(\bar{x}\pm s)/[n(\text{\%})\right]$.

General clinical data		Joint group (*n* = 49)	LC group (*n* = 51)	*t*/χ^2^	*P*
Sex	Male	19	24	0.403	0.700
	Female	30	27		
Average age (years)		49.37 ± 14.00	49.92 ± 15.10	0.189	0.851
Average BMI (kg/m^2^)		24.61 ± 2.92	25.29 ± 3.01	1.146	0.255
Clinical manifestations					
Right upper quadrant pain		49 (100%)	51 (100%)	–	–
Fever (≥38°C)		38 (77.6%)	36 (70.6%)	0.632	0.427
Nausea and/or vomiting		32 (65.3%)	30 (58.8%)	0.456	0.499
Jaundice		12 (24.5%)	10 (19.6%)	0.354	0.552
Right upper quadrant tenderness		49 (100%)	51 (100%)	–	–
Laboratory parameters					
LYM		1.54 ± 0.53	1.72 ± 1.58	0.757	0.451
PLT		235.94 ± 64.48	242.80 ± 59.00	0.555	0.580
Imaging features					
Gallbladder wall thickening (>4 mm)		47 (95.9%)	48 (94.1%)	0.172	0.678
Pericholecystic fluid		38 (77.6%)	35 (68.6%)	1.008	0.315
Gallbladder distension		42 (85.7%)	41 (80.4%)	0.511	0.475
Positive sonographic Murphy sign		43 (87.8%)	44 (86.3%)	0.048	0.827
Systolic blood pressure		132.06 ± 17.40	133.53 ± 22.33	0.366	0.715
Diastolic blood pressure		77.53 ± 11.00	78.12 ± 12.51	0.250	0.803
History of abdominal surgery		6	7	0.826	0.051
Alcohol consumption		0	1	0.325	0.970
Hypertension		10	14	0.410	0.680
Diabetes		2	4	0.428	0.627

### Comparison of surgical parameters

**Figure 2:**
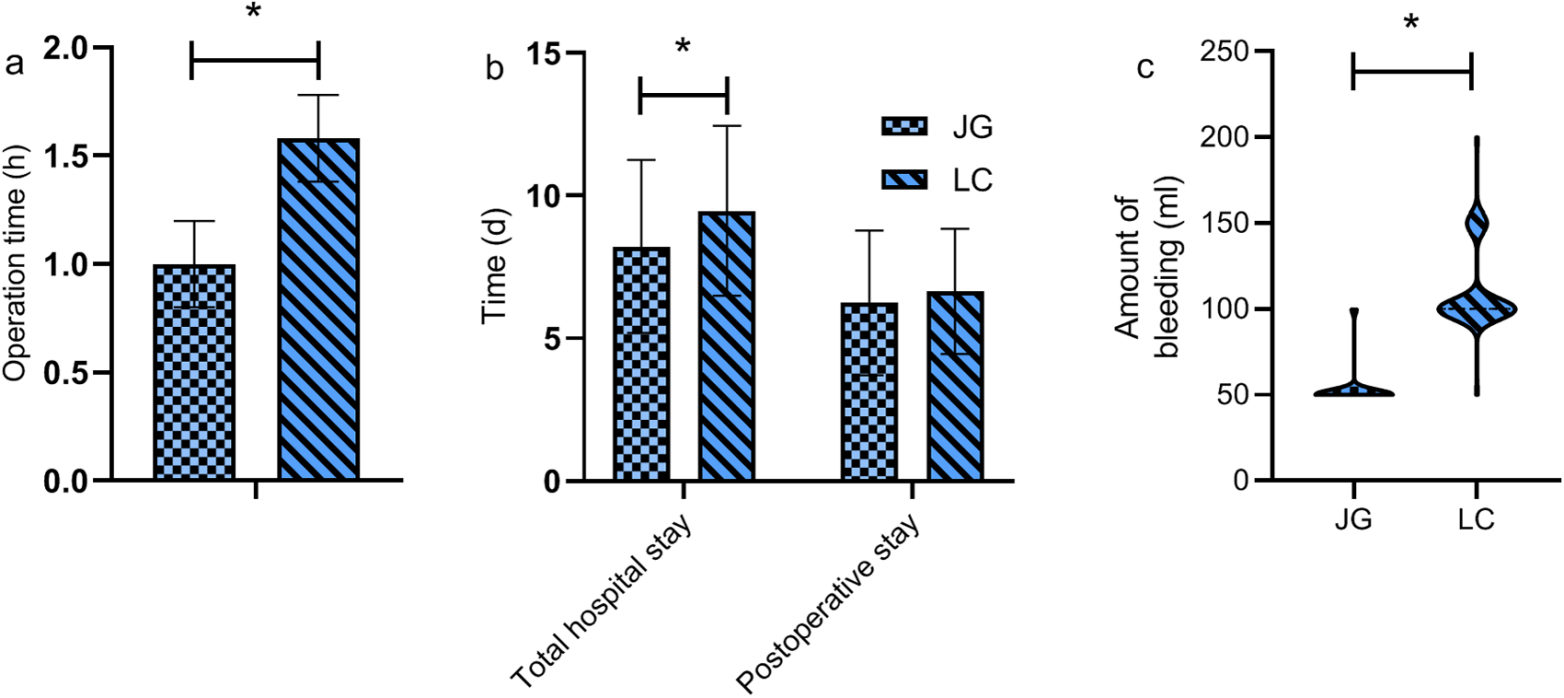
Comparison of surgical parameters between the two groups. The joint group demonstrated a significantly shorter surgery duration (a) and lower intraoperative blood loss (c) compared to the LC group (*P* < 0.05). However, there were no statistically significant differences between the groups in terms of total hospital stay and postoperative stay (*P* > 0.05) (b). Note: JG, joint group; LC, LC group. * indicates a statistically significant difference between groups.

The joint group had a significantly shorter surgery duration and less intraoperative blood loss compared to the LC group (*P* < 0.05). No significant differences were observed in total hospital stay and postoperative stay (*P* > 0.05, Figure 2).

### Comparison of preoperative and 3-day postoperative inflammatory markers

**Figure 3:**
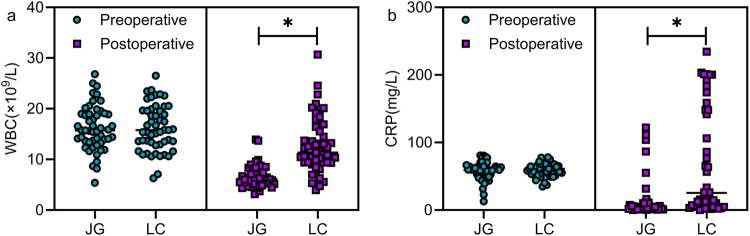
Comparison of preoperative and 3-day postoperative inflammatory markers between the two groups. Preoperative comparison revealed no statistically significant differences in the levels of WBC (a) and CRP (b) between the two groups (*P* > 0.05). On the third postoperative day, patients in the joint group exhibited lower levels of WBC and CRP than those in the LC group (*P* < 0.05). Note: JG, joint group; LC, LC group. * indicates a statistically significant difference between groups.

No significant differences were observed in the levels of preoperative WBC and CRP between the two groups (*P* > 0.05). On postoperative day 3, both markers were significantly lower in the joint group (*P* < 0.05, Figure 3).

### Comparison of preoperative and postoperative liver function indicators

**Figure 4:**
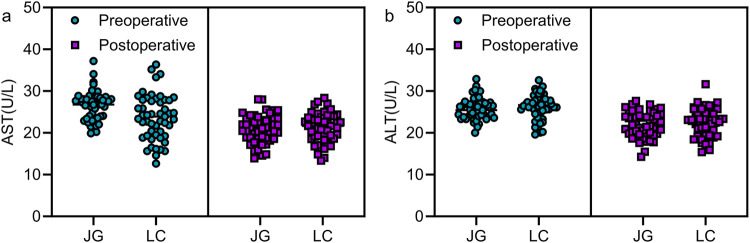
Comparison of preoperative and postoperative liver function indicators between the two groups. No statistically significant differences were observed in AST and ALT levels between the two groups preoperatively and 3 days postoperatively (*P* > 0.05). Note: JG, joint group; LC, LC group.

No significant differences were found in AST and ALT levels between the two groups either preoperatively or on postoperative day 3 (*P* > 0.05, Figure 4).

### Comparison of postoperative complications

The joint group had slightly lower incidences of bile leaks, abdominal infections, incision infections, and bleeding compared to the LC group, but none reached statistical significance (*P* > 0.05, Table 2).

**Table 2:** Comparison of postoperative complication rates between the two groups [*n* (%)].

Group	Number of cases	Bile leaks	Incision infections	Abdominal infections	Bleeding	Total incidence
Joint group	49	0 (0.00)	3 (6.12)	0 (0.00)	2 (4.08)	5 (10.20)
LC group	51	1 (1.96)	4 (7.84)	2 (3.92)	3 (5.88)	10 (19.61)
χ^2^	–	–	–	–	–	1.733
*P*	–	–	–	–	–	0.188

### Comparison of postoperative recovery indicators

**Figure 5:**
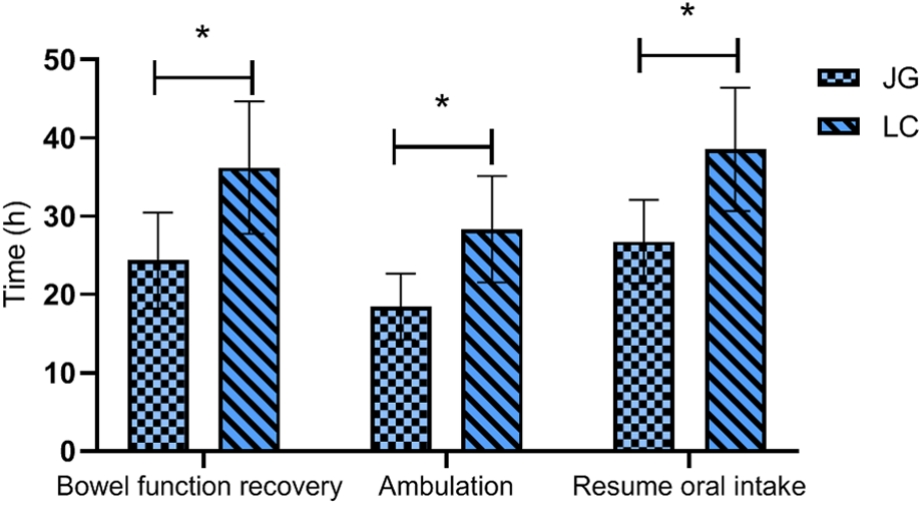
Comparison of postoperative recovery indicators between the two groups. The joint group showed faster recovery of bowel function, earlier ambulation, and shorter time to resume oral intake compared to the LC group postoperatively (*P* < 0.05). Note: JG, joint group; LC, LC group. * indicates a statistically significant difference between groups.

The joint group achieved earlier bowel function recovery, earlier ambulation, and shorter time to resume oral intake compared to the LC group, with all differences reaching statistical significance (*P* < 0.05, Figure 5).

### Comparison of patient satisfaction

**Figure 6:**
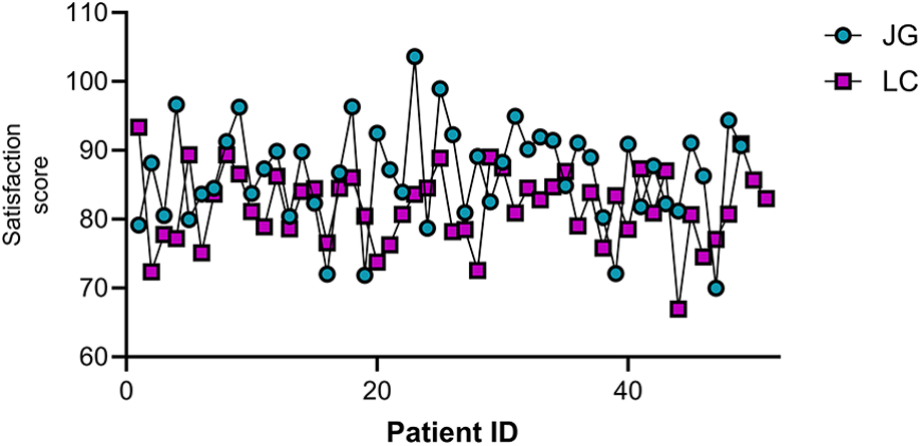
Comparison of patient satisfaction scores between the two groups.

The joint group reported significantly higher satisfaction scores (86.53 ± 7.20) compared to the LC group (81.84 ± 5.40) (*P* < 0.05, Figure 6).

### Comparison of economic indicators

**Figure 7:**
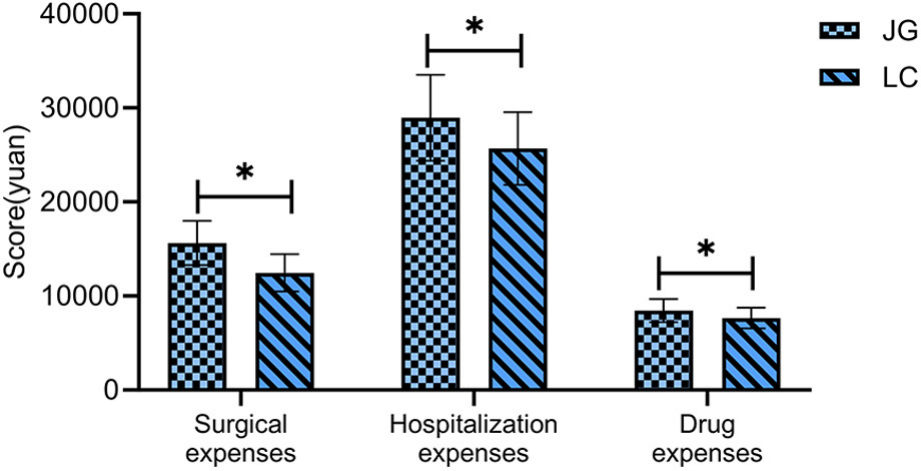
Comparison of economic indicators between the two groups.

The joint group incurred significantly higher surgical costs, total hospitalization costs, and medication costs than the LC group (*P* < 0.05, Figure 7).

### Univariate and multivariable analysis of risk factors for complications

**Figure 8:**
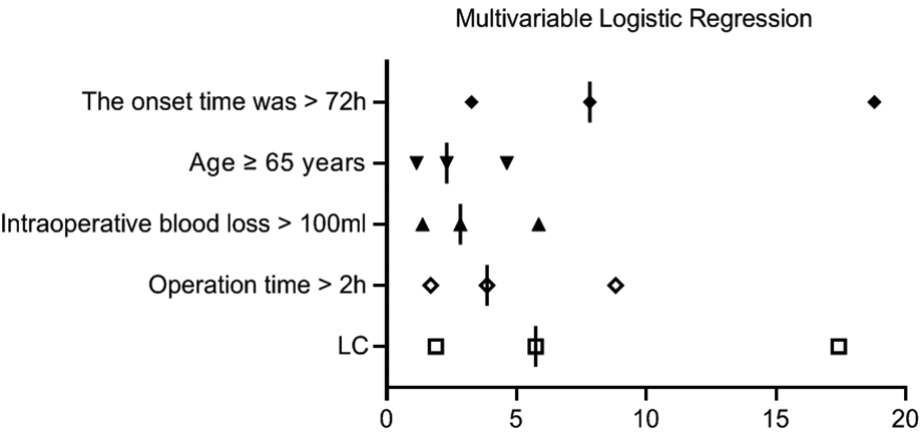
Multivariable logistic regression analysis results of risk factors for postoperative complications.

The presence of postoperative complications was considered the dependent variable (complications present = 0, complications absent = 1), while the independent variables included demographic characteristics (such as sex, age, BMI), underlying diseases (hypertension, diabetes, history of abdominal surgery), disease-related factors (disease onset longer than 72 h, preoperative WBC levels), and surgical-related factors (surgical approach, surgery duration, intraoperative blood loss). Univariate analysis identified age ≥65 years, BMI ≥25 kg/m^2^, hypertension, diabetes, disease onset longer than 72 h, surgical approach (LC alone), surgery duration longer than 2 h, and intraoperative blood loss >100 ml as significant factors of risk factors for postoperative complications (*P* < 0.1, Table 3). Multivariable logistic regression identified disease onset longer than 72 h, surgical approach (LC alone), surgery duration longer than 2 h, intraoperative blood loss >100 ml, and age ≥65 years as independent risk factors for postoperative complications (*P* < 0.05, Table 4, Figure 8).

**Table 3:** Univariate analysis results of risk factors for postoperative complications.

Risk factors		Complications present (*n* = 15)	Complications absent (*n* = 85)	*P*
Demographic characteristics	Age ≥65 years	8 (53.3)	25 (29.4)	0.009
	Sex (male/female)	9/6	34/51	0.325
	BMI ≥25 kg/m^2^	10 (66.7)	38 (44.7)	0.019
Underlying diseases	Hypertension	7 (46.7)	17 (20.0)	0.002
	Diabetes	3 (20.0)	3 (3.5)	0.001
	History of abdominal surgery	5 (33.3)	8 (9.4)	0.225
Disease-related factors	Disease onset longer than 72 h	11 (73.3)	19 (22.4)	<0.001
	WBC >15 × 10^9^/L	9 (60.0)	28 (32.9)	0.156
	NEU >10 × 10^9^/L	8 (53.3)	27 (31.8)	0.229
	CRP >50 mg/L	10 (66.7)	31 (36.5)	0.168
Surgical-related factors	Surgical approach (LC alone)	12 (80.0)	39 (45.9)	<0.001
	Surgery duration longer than 2 h	9 (60.0)	25 (29.4)	0.001
	Intraoperative blood loss > 100 ml	8 (53.3)	22 (25.9)	0.002

**Table 4:** Multivariable logistic regression analysis results of risk factors for postoperative complications.

Risk factors	B	S.E	Wald	OR	95% CI	*P*
Disease onset longer than 72 h	2.058	0.446	21.287	7.83	3.26–18.79	<0.001
Surgical approach (LC alone)	1.747	0.566	9.524	5.74	1.89–17.42	0.002
Surgery duration longer than 2 h	1.351	0.421	10.307	3.86	1.69–8.82	0.001
Intraoperative blood loss >100 ml	1.044	0.369	8.008	2.84	1.38–5.85	0.005
Age ≥65 years	0.837	0.355	5.563	2.31	1.15–4.63	0.018

## Discussion

This study compared the clinical outcomes of LC combined with PTGBD vs. LC alone in treating severe acute cholecystitis. The results showed that the joint group had clear advantages in surgical parameters, including shorter surgery duration and reduced intraoperative blood loss. Additionally, patients in the joint group experienced faster postoperative recovery, greater improvements in inflammatory markers, and higher satisfaction. However, the combined approach was associated with higher hospitalization costs. Several independent risk factors for postoperative complications were identified, including onset of symptoms longer than 72 h, LC alone, surgery duration longer than 2 h, intraoperative blood loss >100 ml, and age ≥65 years.

The observed reductions in surgery duration and blood loss in the joint group are consistent with previous studies. Nassar et al. (9) reviewed 14 studies involving 1,347 cases and found that PTGBD reduced complications, biliary tract injuries, and hospital stay. Similarly, Wang et al. (10) reviewed 12 studies involving 4,379 patients, reporting that the combined approach led to less bleeding, reduced tissue damage, and faster recovery. These findings may be attributable to preoperative drainage through PTGBD, which decreases gallbladder pressure, alleviates inflammation, and improves surgical exposure, ultimately simplifying the procedure (11, 12). Despite these benefits, patients in the joint group had a slightly longer total hospital stay, likely due to the interval required between PTGBD and LC. Subramani et al. (13) highlighted a similar observation in patients with obstructive jaundice, noting that surgery after inflammation resolution minimized complications. This underscores the importance of early anti-inflammatory treatment upon admission, which may explain the longer hospital stay in the joint group.

On postoperative day 3, WBC and CRP levels were significantly lower in the joint group, indicating better control of the inflammatory response. Kang et al. (14) observed similar benefits in high-risk patients treated with PTGD, with a complication rate of about 10.8%. Jin et al. (15) also reported that PTGD led to reduced inflammatory markers, faster recovery, and fewer complications in patients with severe cholecystitis. These improvements likely stem from reduced gallbladder inflammation before surgery, leading to less surgical trauma and a milder postoperative inflammatory response (16, 17). However, our study did not observe significant differences in liver function indicators, possibly due to differences in patient selection or timing of measurements. PTGBD may offer greater benefits for patients with impaired liver function, suggesting the need for individualized surgical planning.

Although the total incidence of complications was slightly lower in the joint group, the difference was not statistically significant. This contrasts with findings from Niiya et al. (18), who reported high technical success and low complication rates with PTGBD. While our study did not show a significant difference, there was a trend toward fewer severe complications, such as bile leaks and abdominal infections, in the joint group. This suggests a potential safety advantage of the combined approach (19), which warrants further validation through large-scale studies.

In terms of postoperative recovery, the joint group showed faster recovery time of bowel function, earlier time to ambulation, and shorter time to resume oral intake. These findings align with Ábrahám et al. (20), who advocated for a staged treatment strategy tailored to individual patient conditions. The reduced surgical trauma in the joint group likely contributed to their quicker recovery. Patient satisfaction scores were also higher in the joint group, probably due to the combination of reduced pain and faster improvement (21).

The study also analyzed the risk factors for postoperative complications. Key predictors included a disease onset longer than 72 h, LC alone, surgery duration longer than 2 h, intraoperative blood loss >100 ml, and age ≥65 years. Notably, a disease onset longer than 72 h emerged as the most significant risk factor (OR = 7.83), emphasizing the need for early intervention. Ie et al. (22) found that a longer interval between disease onset and surgical procedure was associated with higher intraoperative bleeding volume and incidence of postoperative complications (such as wound infection and liver abscess), concluding that prompt surgical intervention significantly improved patient outcomes. LC alone was the second most significant risk factor (OR = 5.74), supporting the value of PTGBD in patients with severe acute cholecystitis. Similar risk indicators were identified by Yamazaki et al. (23), who highlighted blood loss ≥45 ml (OS = 12.14), age ≥75 years, and an ASA-PS score ≥3 (OS = 9.85) as independent risk factors for major perioperative complications. Monitoring these risk factors can help clinicians refine perioperative strategies and develop individualized treatment plans (24).

In terms of economic evaluation, the combined surgical approach was associated with significantly higher surgical, hospitalization, and medication costs. While earlier studies (25) have suggested that the combined approach may reduce long-term healthcare costs by lowering complication and readmission rates, our study lacked long-term follow-up and economic analysis. As such, it is not possible to evaluate the long-term cost-effectiveness of the two surgical strategies. The current findings only demonstrate that the combined procedure is associated with higher direct medical costs during hospitalization. Whether it offers long-term economic benefits remains to be determined through well-designed, prospective studies with adequate follow-up. Clinical decision-making should take into account both the potential clinical benefits and the financial burden on patients, particularly those with limited economic resources.

In conclusion, compared with LC alone, LC combined with PTGBD is more effective in reducing surgical trauma in patients with severe acute cholecystitis, improving postoperative inflammatory markers, and accelerating recovery. However, this combined approach is associated with significantly higher direct medical costs during hospitalization. It is recommended to intensify perioperative intervention for patients of advanced age, those undergoing LC alone, and those with prolonged surgery duration and blood loss, to reduce the incidence of perioperative complications. The strength of this study lies in its systematic evaluation of combined surgery and identification of independent risk factors using multivariable analysis. However, this study has several limitations. First, as a single-center retrospective study, the sample size was relatively limited. Second, the follow-up period was short and restricted to the duration of hospitalization; important outcomes such as 30-day readmission rates and late postoperative complications were not assessed, which may limit the clinical generalizability of the findings. Third, some economic indicators, such as long-term healthcare costs, were not included in the analysis. Future studies should adopt a prospective design, increase the sample size, and extend the follow-up beyond 30 days to allow for a more comprehensive evaluation of the long-term outcomes of the two surgical strategies.

## Data Availability

The raw data supporting the conclusions of this article will be made available by the authors, without undue reservation.
